# Complementary Untargeted and Targeted Metabolomics for Differentiation of Extra Virgin Olive Oils of Different Origin of Purchase Based on Volatile and Phenolic Composition and Sensory Quality

**DOI:** 10.3390/molecules24162896

**Published:** 2019-08-09

**Authors:** Alessio Da Ros, Domenico Masuero, Samantha Riccadonna, Karolina Brkić Bubola, Nadia Mulinacci, Fulvio Mattivi, Igor Lukić, Urska Vrhovsek

**Affiliations:** 1 Fondazione Edmund Mach, Research and Innovation Centre, Via E. Mach, 1 38010 San Michele all’Adige, Trento, Italy; 2 Institute of Agriculture and Tourism, Karla Huguesa 8, 52440 Poreč, Croatia; 3 NEUROFARBA, Pharmaceutical and Nutraceutical Division, University of Florence, Via Ugo Schiff 6, 50019 Sesto Fiorentino, FI, Italy; 4 Department of Physics, University of Trento, Via Sommarive 14, 38123 Povo, TN, Italy

**Keywords:** extra virgin olive oil, volatiles aroma compounds, phenols, sensory attributes

## Abstract

In order to differentiate the extra virgin olive oils (EVOO) of different origin of purchase, such as monovarietal Italian EVOO with protected denomination of origin (PDO) and commercial-blended EVOO purchased in supermarkets, a number of samples was subjected to the analysis of volatile aroma compounds by both targeted gas chromatography/mass spectrometry (GC-MS) and untargeted profiling by comprehensive two-dimensional gas chromatography/mass spectrometry (GC×GC-TOF-MS), analysis of phenols by targeted high-performance liquid chromatography/mass spectrometry (HPLC-DAD-ESI/MS), and quantitative descriptive sensory analysis. Monovarietal PDO EVOOs were characterized by notably higher amounts of positive LOX-derived C6 and C5 volatile compounds, which corresponded to the higher intensities of all the assessed positive fruity and green odor sensory attributes. Commercial-blended EVOOs had larger quantities of generally undesirable esters, alcohols, acids, and aldehydes, which coincided with the occurrence of sensory defects in many samples. Many minor volatile compounds that were identified by GC×GC-TOF-MS were found to differentiate each of the two investigated groups. The differences between the groups with respect to phenols and taste characteristics were evident, but less pronounced. The results that were obtained in this study have undoubtedly confirmed the existence of the large heterogeneity of oils that are sold declared as EVOO. It was shown that GC-MS, GC×GC-TOF-MS, and HPLC-DAD-ESI/MS analyses have complementary outputs, and that their use in combination has advantages in supporting the results of sensory analysis and objectively differentiating these groups of EVOO.

## 1. Introduction

During the previous few decades, the olive oil scientific community and industry have become increasingly linked by the common goal of improving olive oil production and quality [1]. One of the most important permanent aims of this sector is to strengthen and improve the diversification of extra virgin olive oil (EVOO) on the market, because there are still a large number of uninformed consumers who consider olive oil as a standard commodity [2]. The consumers are often unaware of the great heterogeneity with respect to sensory and nutritional quality of oils within the category of the highest quality grade (EVOO), which is mostly present because the thresholds that are set by the European Commission regulation (EEC, 1991) for chemical parameters (acidity, peroxide value, K_232_, K_270_, ΔK values, alkyl esters) are not rigorous and they can be met relatively easily, while the prescribed sensory analysis method practically only discriminates oils with certain fruitiness (median > 0) and without sensory defects from the defective ones [3]. As a consequence, rather different products comply with the requirements of the EVOO category, which often confuses the consumers, especially when it comes down to the large span of EVOO prices [4]. The mentioned heterogeneity of EVOO is strongly influenced by variations in pedoclimatic conditions, cultivar, agronomic practices, and technological factors, and especially by the level of care that is taken in order to avoid or minimize the negative effects from various sources [4,5,6], which is proportional to the costs of production and EVOO final price [4].

Consumers, mostly because of its pleasant aroma, bitterness, and pungency, as well as its health effects, appreciate EVOO. The chemical parameters that are not regulated by the legislation, but they are certainly among the most meaningful for evaluating and understanding EVOO sensory and nutritional quality and they could serve as differentiators that are based on such criteria, are volatile aroma compounds and phenols [7,8,9]. The typical fruity and green aroma of high quality EVOO mainly consists of C5 and C6 aldehydes and ketones, enzymatically formed by lipoxygenase (LOX) and hydroperoxide lyase (HPL) during olive processing, later partly reduced to C6 alcohols by alcohol dehydrogenase (ADH) and then transformed to C6 esters by alcohol acyl transferase (AAT) [10]. In addition, there are other important classes of molecules in EVOO, such as hydrocarbons, acids, and terpenes, which contribute to generating either positive (wood, lemon, rose) or negative notes (rancid, butter, vinegar) [11,12]. Phenols in EVOO are responsible for its taste characteristics, such as bitterness, pungency, and astringency, and they are characterized by a diversity of chemical families, including phenolic alcohols and acids, hydroxy-isochromans, flavonoids, lignans, and secoiridoids. The most abundant among them, secoiridoids, are specific compounds of *Oleaceae* plants and they differentiate olive oil as unique among the other vegetable oils [13,14]. Secoiridoids, more specifically oleuropein glucoside, and its aglycon, are the key contributors to EVOO bitterness [15], although it has been observed that benzoic and cinnamic acid derivatives are also responsible for bitter mouthfeel [5]. EVOO pungency also derives from secoiridoids, especially from the dialdehydic form of decarboxymethylelenolic acid linked to tyrosol, also known as *p*-HPEA-EDA or oleocanthal [16]. EVOO phenols exhibit antioxidant activity which is widely responsible for its oxidative stability and shelf life, as well as its nutritive value [17]. In fact, the European Food Safety Authority (EFSA) allowed for olive oil producers to declare a health claim on the bottle label regarding their positive effect on blood lipids (European Commission, 2012), confirming that the content and composition of phenols can be directly used as an indicator of EVOO quality. To our knowledge, a rather small number of studies investigated the diversity within the EVOO category with respect to various quality indicators [1].

In this case study, two groups of olive oil, declared and sold on the Italian market as EVOO, were compared: i) monovarietal EVOOs with Protected Designation of Origin (PDO) directly purchased from producers and ii) commercial-blended EVOOs that were purchased from national grocery stores (supermarkets). Judging from the anecdotal experience, these two groups, which are frequently the subject of comparison and controversy among experts and consumers regarding their quality and price, should be fundamentally different, but this has not yet been scientifically confirmed to our knowledge. Accordingly, the aim of this study was to differentiate monovarietal PDO and commercial-blended EVOOs on the basis of volatile aroma and phenol composition, and to find reliable indicators of sensory quality among these compounds. It was considered that such findings would significantly contribute to EVOO diversification and they would help to clarify the interrelationship between EVOO origin, quality, and price, and in this way support the growth of the niche in the market segment of consumers informed and interested in healthy, quality products with remarkable diversity.

Apart from being the first that aimed to differentiate EVOO on the basis of the origin of purchase, one of the most important novelties of this study is the utilization of a combined untargeted and targeted metabolomics approach utilizing powerful instrumentation and techniques, such as high throughput two-dimensional gas chromatography with time-of-flight mass spectrometric detection (GC×GC-TOF-MS) complemented by conventional monodimensional GC-MS for the analysis of volatile compounds, and high-performance liquid chromatography with diode array detection and electrospray ionization mass spectrometry (HPLC-DAD-ESI-MS) for the analysis of phenols. As a result, this study reported one of the most detailed and comprehensive qualitative and quantitative analytical characterizations of the volatile profile in EVOO up to date, with many compounds being identified (or tentatively identified) in EVOO for the first time. Additionally, it provided novel evidence regarding the diversity in sensory quality and volatile composition of olive oils sold declared as pertaining to the category of the highest quality grade (EVOO) and confirmed the need to re-evaluate the categorization criteria that were set by the current legislation.

## 2. Results and Discussion

### 2.1. Sensory Attributes

**Figure 1:**
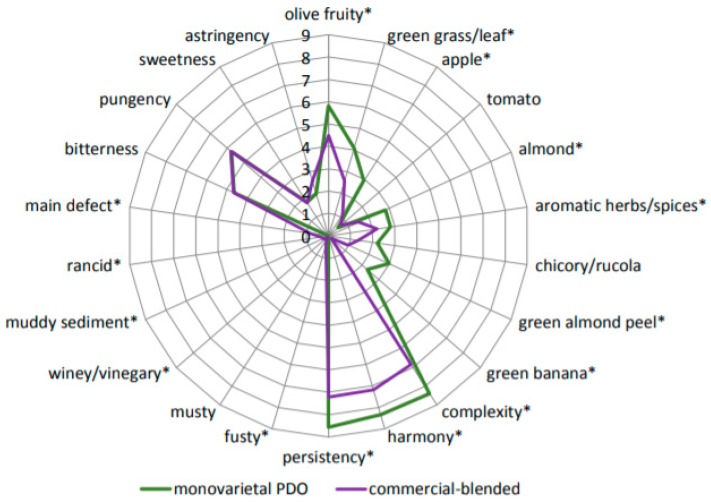
Spiderweb diagram with statistical significance differences indicated by (*) asterisk for each sensory attribute and defect.

The results of sensory analysis are reported in a spider-web diagram in Figure 1. Monovarietal PDO EVOOs were characterized by higher intensities of the majority of positive odor descriptors, such as apple, green grass/leaf, aromatic herbs, etc. (except tomato and chicory/rucola), as well as general hedonic attributes, such as harmony, complexity, and persistency. Mild intensities of various sensory defects were detected in 19 out of 25 commercial-blended EVOO samples, which cast doubt on the correctness of their categorization and declaration. The average values of each defect intensity should be interpreted with caution, since not all of the samples had the same defect. For this reason, the average of the main perceived defect (the defect with the highest intensity perceived in each sample) was also calculated and is presented in Figure 1. No defects were detected in the monovarietal PDO samples. Interestingly, no significant differences were found for the taste attributes.

### 2.2. Volatile Compounds (VOCs) and Sensory Attributes

#### 2.2.1. GC-MS and Sensory Attributes

The characteristic and unique flavor of EVOO, in particular its green and fruity attributes depend on many volatile compounds [10,11]. The identification and quantification of the compounds, causing both positive odors and off-flavors, is considered to be crucial for EVOO quality control. The list of selected identified and confirmed compounds, sorted by decreasing *F*-value, is shown in Table 1. Two groups of samples were successfully differentiated by one-way ANOVA. The concentrations of the majority of C6 and C5 aldehydes, which are regularly listed among the key ones that are responsible for positive green and fruity odors [18,19], were clearly higher in monovarietal PDO EVOOs than in the commercial-blended ones. The most abundant volatile compound in all of the investigated samples, (*E*)-2-hexanal, was also found in higher amounts in monovarietal PDO EVOOs, although it was not among the ones with the highest discriminative power judging from the *F*-values. However, (*Z*)-2-hexenal and (*Z*)-3-hexenal, carriers of major positive fruity and green notes, together with the isomers of 3-ethyl-1,5-octadiene and 4,8-dimethyl-1,3,7-nonatriene with unknown sensory relevance [20], turned out to be decisive for the differentiation of monovarietal PDO from commercial blended EVOOs. Among other possible causes of the lower amounts of these compounds in commercial blended EVOOs were possibly the changes induced by EVOO aging during storage, as it was shown in earlier studies [21]. In contrast to monovarietal PDO EVOOs that were analyzed relatively fresh, the age of commercial blended EVOO was not declared by the producers/sellers and it was practically unknown, and it was possible that these samples were produced or partially composed from olive oils that were obtained in harvests prior to 2016. Nevertheless, it must be kept in mind that all of the investigated EVOOs were carefully selected and sampled at the same time and they were therefore valid and authentic representatives of both groups of EVOOs offered on the market at that given moment [21].

The commercial-blended EVOOs from supermarkets were mostly characterized by the higher concentration of saturated esters, aldehydes, and alcohols (Table 1). Such compounds do not originate from the LOX pathway and they are mostly the result of other, mostly undesirable processes [21,22,23]. Ethyl and methyl acetate, which are responsible for winey-vinegar defect and, together with 2-methylbutanol and 2-phenylethanol, clearly indicated that olives underwent fermentation [12], were the major differentiators of the commercial-blended from the monovarietal PDO EVOOs. Non-LOX C4 and C5 branched compounds are known to derive from the conversion of certain amino acids, while linear acids, esters, and ketones originate from fatty acid metabolism [24]. All of the mentioned processes are commonly linked to more or less degraded raw olive fruit material, due to physical damage, inadequate sanitary conditions, or unsuitable storage of fruit before processing, and they are often found in VOOs with sensory defects [24,25,26,27]. The possibility that particular non-LOX volatile compounds were formed and/or increased in concentration as a result of various oxidative processes during an (unknown) storage period of a number of commercial blended EVOOs, as shown previously by other authors [21], should not be neglected.

**Figure 2:**
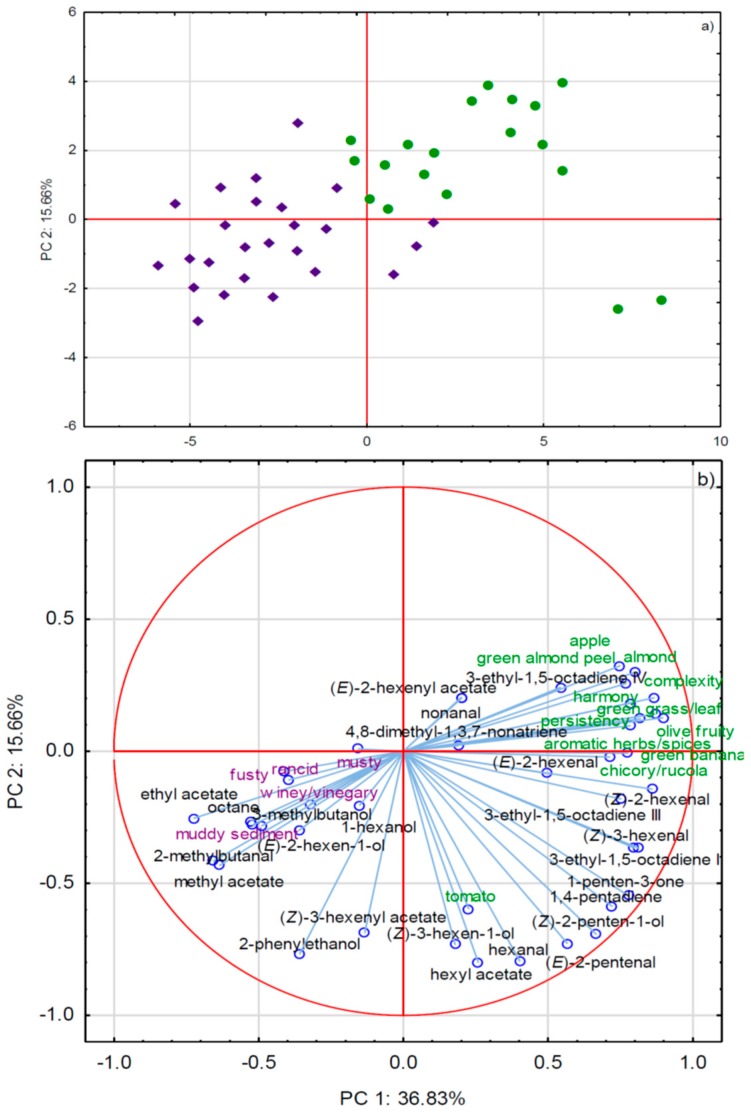
(**a**) Separation of olive oil samples sold as extra virgin olive oil (EVOO) in Italy according to the origin of purchase by the first two principal components, PC1 and PC2. Green cycles represent monovarietal PDO EVOO purchased on family farms, while violet rhombs represent commercial-blended EVOO purchased in supermarkets. (**b**) Factor loadings of selected variables, i.e., concentrations of volatile aroma compounds and intensities of sensory attributes, obtained by GC-MS and sensory analyses, respectively, on PC1 and PC2.

PCA analysis clearly divided the samples in two groups (Figure 2). The majority of the investigated aldehydes and ketones that derive from the LOX pathway, including the major ones, such as (*E*)-2-, (*Z*)-2-, and (*Z*)-3-hexenal, as well as 1-penten-3-one and unsaturated hydrocarbons, were characteristic for monovarietal PDO EVOO samples, and they could have contributed to generating positive green and fruity notes [19] since a positive correlation between their concentrations and the intensities of such sensory attributes was evident (Figure 2b). Sensory defects that were observed in the commercial-blended EVOOs most probably, at least partly, originated from the elevated concentrations of fermentation and oxidation derived volatiles, such as linear alcohols and esters (Figure 2).

#### 2.2.2. GC×GC-TOF-MS and Sensory Attributes

The first preliminary classification of monovarietal PDO and commercial-blended EVOO samples that were based on untargeted profiling information was obtained by applying PCA on raw data. However, not only selected bidimensional GC peaks with the highest potential for varietal differentiation, but also other peaks were tentatively identified on the basis of mass spectrum and linear retention index matching to improve its effectiveness and specificity and to obtain as much qualitative information as possible. Table 2 reports the list of volatile aroma compounds tentatively annotated in the investigated EVOOs after GC×GC-TOF-MS analysis, in order of decreasing *F*-ratio.

**Figure 3:**
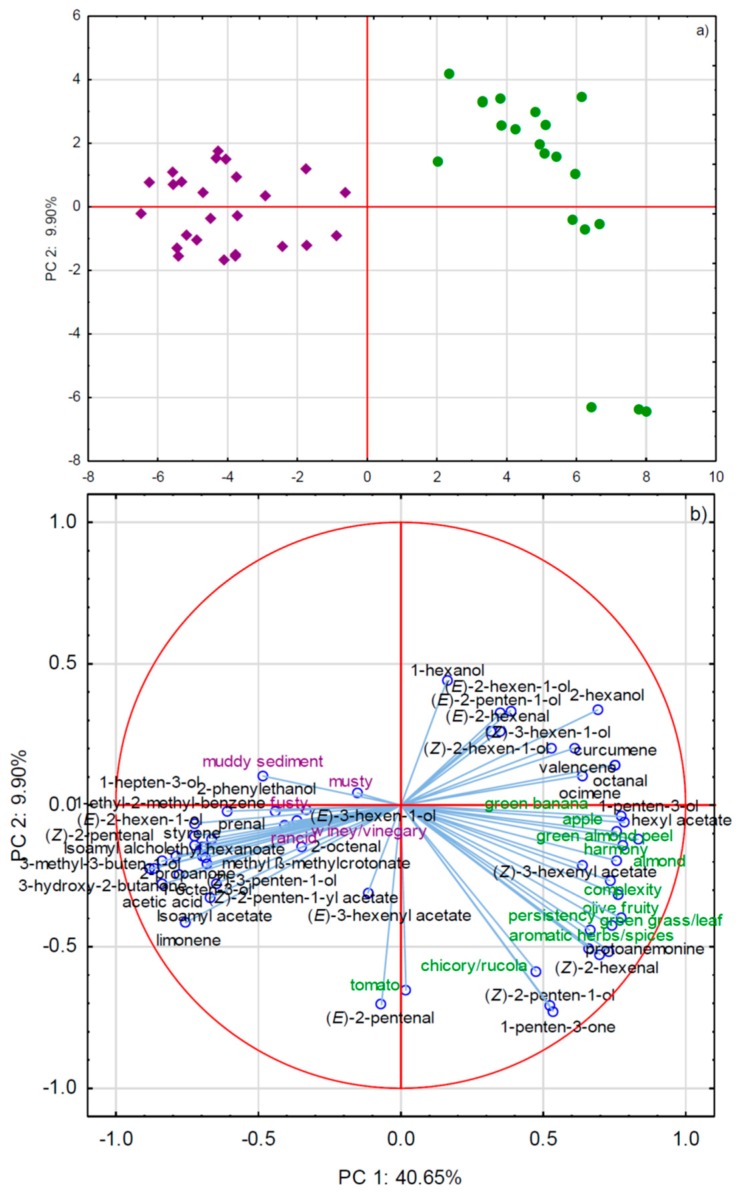
(**a**) Separation of olive oils sold as extra virgin olive oil (EVOO) in Italy according to the origin of purchase in two-dimensional space defined by the first two principal components, PC1 and PC2. Green cycles represent monovarietal PDO EVOO purchased on family farms, while violet rhombs represent commercial-blended EVOO purchased in supermarkets (**b**) Factor loadings of selected variables, i.e., concentrations of volatile aroma compounds and intensities of sensory attributes, obtained by GC×GC-TOF-MS and sensory analyses, respectively, on PC1 and PC2.

GC×GC-TOF-MS analysis extracted many minor compounds as statistically relevant for this study, which were not previously identified by GC-MS, either in this or in earlier studies. Such a result proved that the two techniques have complementary outputs and that their use in combination has serious advantages. Interestingly, several compounds that were most characteristic (the highest *F*-values) for monovarietal PDO EVOOs were the minor ones, such as curcumene, octanal, 2-hexanol, ocimene, protoamenonine, valencene, etc. (Table 2). On the other hand, GC×GC-TOF-MS in a major part confirmed the GC-MS results, among other findings that higher amounts of standardly reported major LOX-derived C6 and C5 aldehydes, ketones, and alcohols, such as (*Z*)-2-hexenal, (*E*)-2-hexenal, 1-penten-3-one, and (*Z*)-2-penten-1-ol, are characteristic for this group of EVOO. The commercial-blended EVOO group was distinguished by a much larger number of volatile markers, including many of those often co-occurring with negative, defective sensory notes, such as 2-phenyl ethanol, isoamyl alcohol, isoamyl acetate, ethyl-2-methyl benzene, ethyl hexanoate, 3-methyl-2-buten-1-ol, 3-hydroxy-2-butanone, etc. [19,21,22,28], accompanied by a large array of minor and unbeknown compounds (Table 2). The most characteristic compounds, such as acetic acid, 3-methyl-3-buten-1-ol, 3-hydroxy-2-butanone, 1-octen-3-ol, and others, did not coincide with those that were extracted as the most important by GC-MS (e.g., acetates), showing once again the synergistic potential of the two techniques used. Again, the group of commercial-blended EVOO was seriously deficient in the LOX volatiles that were known to be carriers of positive green and olive fruity flavor notes. Such results were confirmed by PCA: monovarietal PDO EVOOs were sharply separated from the commercial-blended group, owing to the higher amounts of LOX volatiles and more intense positive odor sensory notes (Figure 3). Although produced from different olive cultivars grown in different geographical areas in Italy, monovarietal PDO EVOOs exhibited a relatively high level of homogeneity, with the exception of the three samples that belonged to the same cultivar/area located in the fourth quadrant of Cartesian plane with high absolute values of the coordinates on both PC1 and PC2 axes, which were even more discriminated from the commercial-blended EVOOs (Figure 3a). It is probable that, in this particular case, the cultivar and/or the geographical area had a greater impact. The commercial-blended EVOOs were mostly grouped by the non-LOX compounds and the occurrence of sensory defects, which were probably in a causal relationship (Figure 3). Indeed, the defects that were observed during the sensory analysis of the commercial EVOOs put these oils in a lower quality category that usually have a lower price than EVOO. The results that were obtained in this study for the particular major volatile compounds were mostly in agreement with the findings from Fiorini et al. (2018) [1], who also utilized their amounts, among other compounds, to differentiate high from low-priced EVOO.

### 2.3. Phenols and Sensory Attributes

**Figure 4:**
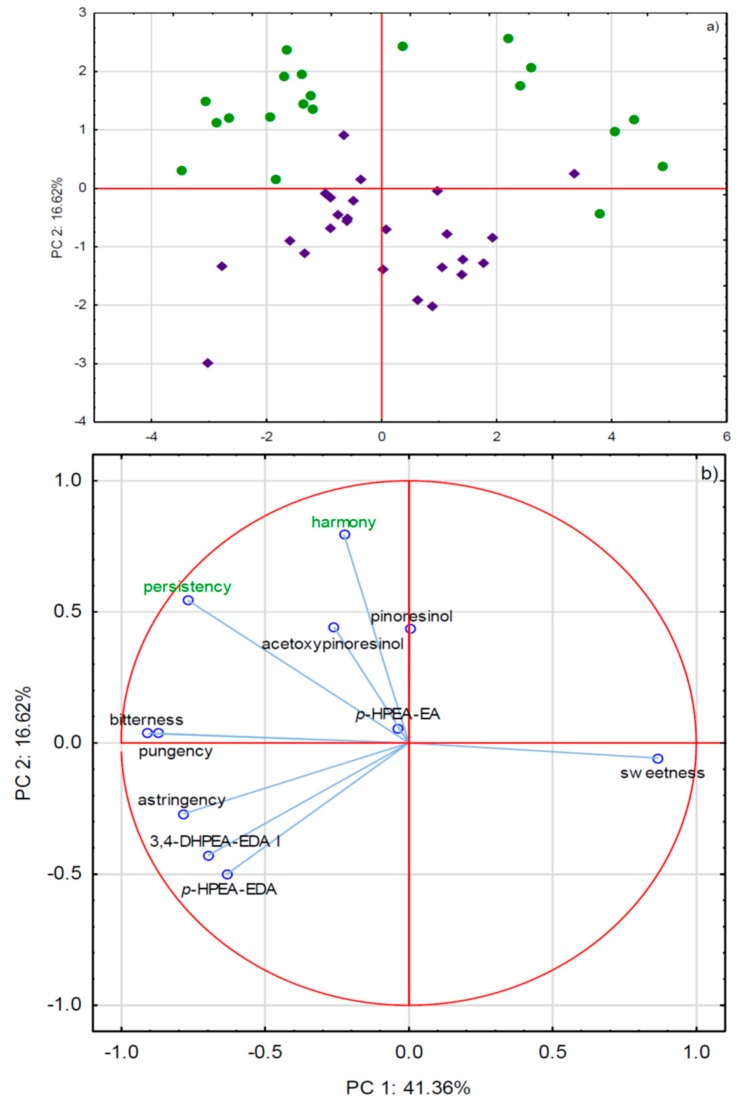
(**a**) Separation of olive oils sold as extra virgin olive oil (EVOO) in Italy according to the origin of purchase in two-dimensional space defined by the first two principal components, PC1 and PC2. Green cycles represent monovarietal PDO EVOO purchased on family farms, while violet rhombs represent commercial-blended EVOO purchased in supermarkets (**b**) Factor loadings of selected variables, i.e., concentrations of phenols and intensities of sensory attributes, obtained by HPLC-DAD-ESI/MS and sensory analyses, respectively, on PC1 and PC2.

In all of the investigated EVOO samples, 13 phenols were identified and quantified (Table 3) while using a wavelength of 310 nm for vanillin and *p*-coumaric acid, while the wavelength was 280 nm for all other compounds. Moreover, the molecular ions of each compound were used to confirm the identification of the analytes. Generally, the most abundant was *p*-HPEA-EDA, followed by 3,4-DHPEA-EDA I, 3,4-DHPEA-EA, *p*-HPEA-EA, and OH-tyrosol. Interestingly, a statistically significant difference was only found for a few phenols: lignans, such as pinoresinol and acetoxypinoresinol, were characteristic for the monovarietal PDO EVOOs, while the commercial-blended group was distinguished by higher amount of *p*-HPEA-EDA. The reduced data matrix that was obtained through HPLC-DAD-ESI/MS quantitative analysis (the phenols with 0.05 *< p* and 0.05 < *p*<0.10) was subjected to PCA (Figure 4). The two classes of EVOO samples were clearly separated in the score plot (Figure 4a). The commercial-blended ones appeared to be relatively homogeneous in terms of phenol composition, since they were grouped rather close in the center of the plot. Monovarietal PDO EVOO samples were scattered in all four quadrants of the Carthesian plane, which indicated a higher degree of diversity. This was even more obvious when all of the phenols were included as variables, resulting with an additional separation of three-sample clusters of particular monovarietal EVOO in PCA representation (Figure S1). Positive sensory descriptors that were partly related to the EVOO taste, such as harmony and persistency, were characteristic for monovarietal PDO EVOOs (Figure 4), although there was no evidence that the phenols were responsible for that. In fact, for the intensities of attributes that are known to directly originate from phenols, such as bitterness, pungency, and astringency, no notable differences were observed between the two groups of EVOO samples. This result is not completely in accordance with the findings from Fiorini et al. (2018) [1], who found high-priced EVOO to be more abundant in the majority of phenols, including secoiridoids, and characterized by higher intensities of bitterness and pungency in relation to EVOO samples of low price.

## 3. Materials and Methods

### 3.1. EVOO Samples

After preliminary selection from a larger group of high quality monovarietal EVOOs with PDO, samples that were produced from olives of Italian cultivars harvested in 2016 were collected from different geographical areas in Italy (price range from 20 to 30 €/L), including Reggio Calabria (cultivar: Ottobratica; *n = 3*), Perugia (cultivar: Moraiolo; *n = 3*), Ragusa (cultivar: Tonda Iblea; *n = 3*), Grosseto (cultivar: Frantoio; *n = 3*), Imperia (cultivar: Taggiasca; *n = 1*), Brescia (cultivar: Moraiolo; *n = 1*), Verona (cultivar: Leccino; *n = 1*), and Riva del Garda (cultivar: Casaliva; *n = 3*). Furthermore, 25 commercial-blended EVOOs (price range from 3 to 12 €/L) were purchased from Italian grocery stores (supermarkets), which were selected according to Nielsen (New York, NY, USA 2016) data as among the most consumed during 2016 in Italy. All of the samples were stored in dark bottles at a controlled temperature of 15 °C before analysis, and gaseous N_2_ was added in the headspace to prevent oxidation each time that the bottles were opened.

### 3.2. Standards and Solvents 

The solvents used for the analysis of phenols in EVOOs were HPLC-MS grade methanol, hexane, isopropanol, and formic acid, which were purchased from Honeywell Riedel-de Haën (Seelze, Germany) and all aqueous solutions, including the HPLC mobile phase, were prepared with water purified while using a Milli-Q system (Millipore, Vimodrone, Milan, Italy). All of the analytical standards used for identification and calibration are listed in Table S1.

### 3.3. GC-MS Analysis of Volatile Aroma Compounds

Three grams of EVOO were put into a 20 mL glass headspace vial, and then spiked with 30 μL of internal standard solution (menthol at 0.057 mg/g; *w*/*w* in seeds oil). The headspace in the vial was equilibrated at 40 °C for 5 min. and the volatile aroma compounds were extracted at 40 °C for 30 min. The headspace was sampled using 2 cm divinylbenzene/carboxen/polydimethylsiloxane (DVB/CAR/PDMS) 50/30 μm fibre, purchased from Supelco (Bellefonte, PA, USA). The volatile aroma compounds were desorbed in the GC inlet at 250 °C for 4 min. in splitless mode, and the fibre was reconditioned for 7 min. at 270 °C, prior to each analysis. Measurements were made while using a Thermo Trace GC Ultra gas chromatograph coupled to a Thermo Quantum XLS mass spectrometer Thermo Scientific (Milan, Italy), which was equipped with a PAL combi-xt (CTC, Zwingen, Switzerland) autosampler with a SPME option. A VF-wax capillary column (30 m × 0.25 mm × 0.25 μm, Agilent Technologies) was used. The GC oven temperature gradient was starting from 40 °C for 4 min., 6 °C/min. up to 250 °C, and held for 5 min. Carrier gas was helium at the constant flow rate of 1.2 mL/min. The transfer line and the MS ion source were both set at 250 °C. Electron ionization was applied at 70 eV with an emission current of 50 mA. Mass spectra were recorded in centroid full scan mode at a scan time of 0.200 s from 30 to 350 *m*/*z*. Thermo Excalibur software (2.2 SP1. 48, Thermo Scientific) was used for all acquisition control and data processing. Figure S1 reports representative chromatograms. Volatile aroma compounds were identified by comparing the retention times and mass spectra with those of standards, and with mass spectra from NIST 2.0, Wiley 8, and FFNSC 2 (Chromaleont, Messina, Italy). Linear retention indexes (relative to C7-C24 *n*-alkanes) were calculated and then compared to those from the literature. Semi-quantitative analysis was carried out and the concentrations of EVOO volatile aroma compounds were expressed as equivalents of the internal standard menthol in mg/kg of oil.

### 3.4. GC×GC-TOF-MS Analysis of Volatile Aroma Compounds

For GC×GC-TOF-MS analysis, a Gerstel MultiPurpose Sampler autosampler (Gerstel GmbH & Co. KG Mülheim an der Ruhr, Germany) with an agitator and SPME fiber was used to extract the volatiles from the EVOO sample vial headspace. The GC×GC system consisted of an Agilent 7890 A (Agilent Technologies, Santa Clara, CA, USA) that was equipped with a Pegasus IV time-of-flight mass spectrometer (Leco Corporation, St. Joseph, MI, USA). A VF-Wax column (100% polyethylene glycol) 30 m × 0.25 mm × 0.25 μm (Agilent J&W Scientific Inc., Folsom, CA, USA) was used as a first-dimension (1D) column, and a RTX-200MS-column 1.50 m × 0.25 mm × 0.25 μm (Restek, Bellefonte, PA, USA) was used as a second-dimension (2D) column. The GC system was equipped with a secondary column oven and a non-moving quadjet dual-stage thermal modulator. The injector/transfer line was maintained at 250 °C. The oven temperature program conditions were as follows: initial temperature of 40 °C for 4 min., programmed at 6 °C/min. up to 250 °C, where it remained for 5 min. The secondary oven was kept 5 °C above the primary oven throughout the chromatographic run. The modulator was offset by +15 °C in relation to the secondary oven; the modulation time was 7 s and 1.4 s of hot pulse duration. Such a modulation optimization was adapted for the analysis of minor volatiles. Helium (99.9995% purity) was used as carrier gas at a constant flow of 1.2 mL/min. The MS parameters included electron ionization at 70 eV with the ion source temperature at 230 °C, detector voltage of 1317 V, mass range of *m*/*z* 35–450, and acquisition rate of 200 spectra/s. Representative contour plots (2D-chromatograms) are reported in Figure S2.

For GC×GC-TOF-MS data, LECO ChromaTOF Version 4.22 software was used for all acquisition control and data processing. Automated peak detection and spectral deconvolution with a baseline offset of 0.8 and signal-to-noise of 100 were used during data treatment. With these settings, it was possible to detect 1479 putative compounds. The identification of VOO volatile aroma compounds was performed by comparing the retention times and mass spectra with those of the pure standards, and with mass spectra from NIST 2.0, Wiley 8, and FFNSC 2 (Chromaleont, Messina, Italy) mass spectral libraries, with a minimum library similarity match factor of 750. Additional identification of volatiles was achieved by comparing the experimental linear temperature retention indices with those that were reported in the literature for 1D-GC. In total, 179 volatile aroma compounds were identified. To account for possible sample-to-sample variation, all of the intensities were normalized to the signal of menthol (internal standard) and corrected for the mass added. The analyses were performed in triplicates, and the average values were used in further data elaboration.

### 3.5. HPLC-DAD-MS Analysis Of Phenols

Samples extraction was made according to [29]. Five grams of oil containing a fixed aliquot of IS (syringic acid = 291 mg/L in MeOH) were dissolved in 5 mL of hexane and then extracted with 5 mL of a methanol: water solution (60:40, *v/v*) five times. Afterwards, 10 mL of hexane were added to the methanolic extracted solution, vortexed, and centrifuged for 5 min. at 5000 rpm. The methanol solution was collected and evaporated to dryness under vacuum. The extract was reconstituted with 2.5 mL of HPLC-grade methanol and then filtered through a 0.22 µm mPTFE filter before HPLC-DAD-MS analysis. 

HPLC-DAD-ESI/MS studies were performed while using an Alliance 2695 HPLC with a diode–array detector (DAD 2996) and a mass spectrometer detector qDa MS, Waters (Milford, MA, USA). The separation was achieved on a Synergi Polar reverse phase (RP) (250 × 4.6 mm, 4 µm) analytical column from Phenomenex (Chesire, UK). The mobile phase for HPLC-DAD-ESI/MS analyzes was water with 0.1% formic acid (A) and methanol/isopropanol solution (90:10 *v*/*v*) with 0.1% formic acid (B) working in the gradient mode at a flow rate of 1 mL min−1. The solvent composition varied, as follows: 0 min., 30% B; 0–40 min., 60% B; 40–53 min. 95% B; 53.1–60 min. 30% B; then, the column was reconditioned. The column temperature was set at 35 °C and the injection volume was 10 µL [29]. Representative chromatograms are reported in Figure S3.

Phenols were quantified based on calibration curves of standards when available, while others were expressed as equivalents: 3,4-DHPEA-EDA-1 (as OH-tyrosol), 3,4-DHPEA-EDA-2 (as OH-tyrosol), 3,4-DHPEA-EA (as OH-tyrosol), *p*-HPEA-EDA (as tyrosol), *p*-HPEA-EA (as tyrosol), and acetoxypinoresinol (as pinoresinol).

### 3.6. Sensory Analysis

Quantitative descriptive analysis of monovarietal and commercial EVOO samples was performed by the VOO sensory analysis panel comprised of eight assessors (four female, four male) that were trained for VOO sensory analysis according to the method that was proposed by IOC described in the European Commission Regulation [30]. The panel is accredited according to the EN ISO/IEC 17025:2007 standard from 2012 and continuously recognized by the IOC from 2014 to December 2019.

The panel used a modified profile sheet that was expanded with particular positive odor and taste attributes [30]. Single odor and taste attributes were quantified while using a 10-cm unstructured intensity ordinal rating scale from 0 (no perception) to 10 (the highest intensity). Differently from the standard method, for evaluating different general hedonic quality attributes (complexity, harmony, and persistency of VOO samples), a 10-point overall structured rating scale from 0 (the lowest quality) to 10 (the highest quality) was applied. For overall quality evaluation, the VOOs were graded with points from 1 (the lowest quality) to 9 (the highest quality).

### 3.7. Statistical Data Elaboration

GC-MS, GC×GC-TOF-MS, HPLC-DAD-ESI/MS, and sensory analyses data (concentrations of volatile aroma compounds and phenols) were subjected to one-way analysis of variance (ANOVA), and the average values were compared by Least Significant Difference (LSD) test at the level of *p* < 0.05. These data, together with the results of sensory analysis (medians of intensities and grades), were further processed by principal component analysis (PCA) in order to better visualize the differences between the two groups of EVOO and explain them on the basis of the concentrations of volatiles and phenols. Three datasets (GC-MS, GC×GC-TOF-MS, and HPLC-DAD-ESI/MS) were separately treated. Prior to PCA analysis, the GC-MS and GC×GC-TOF-MS original datasets were reduced to only include the volatiles with the highest discriminative potency (*F*-values in one-way ANOVA), combined with those regularly reported as among the most important for EVOO aroma. For the HPLC-DAD-ESI/MS dataset, first PCA was performed including all the identified phenols (Figure S4), and then it was applied on a reduced dataset including only those for which statistically significant differences were observed (*p* < 0.05), and those being close to that (0.05 < *p* < 0.10). Statistical data elaboration was performed by Statistica v. 13.2 software (Stat-Soft Inc., Tulsa, OK, USA).

## 4. Conclusions

The combined use of GC-MS and GC×GC-TOF-MS analysis for volatile aroma compounds proved to be a powerful analytical option, providing broad coverage of the volatilome, which is useful for the differentiation of the two classes of EVOO with respect to the origin of purchase: monovarietal PDO EVOO from family farms vs. commercial-blended EVOO from supermarkets, respectively. To our knowledge, this study provided one of the most detailed and comprehensive qualitative and quantitative analytical characterizations of the volatile profile in EVOO up to date, with many compounds being identified (or tentatively identified) in EVOO for the first time. Among them, many potential markers were extracted, despite the known (for PDO) and presumed (for commercial-blended) geographical and pedoclimatic heterogeneity and large variations in olive growing and oil producing parameters among EVOOs. Monovarietal PDO EVOOs were characterized by notably higher concentrations of desirable LOX-derived C6 and C5 volatiles, including the major ones that are known to be crucial contributors to the characteristic and appreciated EVOO green and fruity flavor. Such findings basically confirmed the results of the sensory analysis, which described the monovarietal PDO EVOOs by higher intensities and grades for positive sensory descriptors and attributes. On the other hand, the commercial-blended EVOOs had larger quantities of many volatiles that are known to originate from undesirable chemical and microbiological processes in olive and in olive oil, such as saturated esters, alcohols, acids, and aldehydes, which corresponded to the occurrence of sensory defects in many of the samples from this group. It is worth highlighting that a very large array of minor and unbeknown compounds, not reported or neglected in previous studies, was found to differentiate each of the two investigated EVOO groups, which point to the possibility that they also contributed to the perceived sensory notes. Targeted HPLC-DAD-ESI/MS profiling of phenols succeeded in differentiating monovarietal PDO from commercial-blended EVOOs to a much smaller extent, which was mostly due to the diversity of the concentration in monovarietal oils. Additionally, the differences that were observed during sensory analysis related to the corresponding taste attributes were not large. Nevertheless, the results of this study undoubtedly confirmed the large heterogeneity of oils, which are sold declared as EVOO in Italy, both in terms of chemical composition and sensory attributes, and in a way pointed to the possible reasons behind the existing large span of prices within this quality category.

The approach that was proposed in this study is universal in nature and it could be applied for the characterization and differentiation of various other types of EVOO. The detailed profiles of volatile aroma compounds and phenols that can be obtained by the reported combination of powerful yet complementary techniques may serve experts, producers, and suppliers to better define typical sensory characteristics of EVOO in question, and in this way strengthen their identity and position on the market. From the technological point of view, understanding the compositional origins of the sensory typicity of particular EVOO might allow for more efficient quality management and control in production and more precise information to the consumers.

## Figures and Tables

**Table 1:** List of volatile aroma compounds found in monovarietal Protected Designation of Origin (PDO) and commercial-blended extra virgin olive oils by headspace solid-phase microextraction combined with gas chromatography/mass spectrometry (HS-SPME-GC-MS) sorted by descending Fisher *F*-ratio, compound class, type of identification, and semi-quantitative values in mg/kg relative to internal standard.

Compounds	Class	Confirmed by	*F*-Ratio	Class	
				Monovarietal	Commercial/Blended
Ethyl acetate	Ester	Std; MS	95.76	0.638	5.465 *
Methyl acetate	Ester	Std; MS	80.37	0.436	2.994 *
3-Ethyl-1,5-octadiene III	Hydrocarbon	MS	64.32	4.260 *	1.853
2-Methylbutanal	Aldehyde	Std; MS	38.87	0.181	0.715 *
3-Ethyl-1,5-octadiene I	Hydrocarbon	MS	37.64	0.957 *	0.511
(*Z*)-2-Hexenal	Aldehyde	Std; MS	27.14	1.696 *	0.591
3-Methylbutanol	Alcohol	Std; MS	22.79	0.477	1.229 *
2-Phenylethanol	Alcohol	Std; MS	18.50	0.181	0.376 *
3-Ethyl-1,5-octadiene IV	Hydrocarbon	MS	17.12	1.434 *	0.473
(*Z*)-3-Hexenal	Aldehyde	Std; MS	15.79	0.844 *	0.320
Octane	Alkane	Std; MS	14.06	1.197	2.912 *
1,4-Pentadiene	Hydrocarbon	MS	12.88	2.889 *	1.582
4,8-Dimethyl-1,3,7-nonatriene	Hydrocarbon	Std; MS	11.60	0.677 *	0.235
1-Penten-3-one	Ketone	Std; MS	11.26	6.508 *	1.772
(*E*)-2-Hexen-1-ol	Alcohol	Std; MS	7.79	4.145	6.825 *
(*E*)-2-Hexenal	Aldehyde	Std; MS	7.79	72.229 *	46.644
(*Z*)-2-Penten-1-ol	Alcohol	RI	4.78	2.413 *	1.570
(*E*)-2-Hexenyl acetate	Ester	Std; MS	n.s	0.249	0.100
(*E*)-2-Pentenal	Aldehyde	Std; MS	n.s	0.708	0.547
(*Z*)-3-Hexen-1-ol	Alcohol	Std; MS	n.s	9.490	8.718
(*Z*)-3-Hexenyl acetate	Ester	Std; MS	n.s	3.184	4.829
1-Hexanol	Alcohol	Std; MS	n.s	6.859	7.009
Hexanal	Aldehyde	Std; MS	n.s	6.535	6.086
Hexyl acetate	Ester	Std; MS	n.s	0.812	0.814
Nonanal	Aldehyde	RI	n.s	0.414	0.149

Identification: the volatile aroma compounds were identified on the basis of the comparison of their mass spectra and retention time with those of pure standards or with mass spectra from a mass spectral database (Std, MS), as well as by retention indexes (RI) matches on a similar phase column (NIST Chemistry WebBook SRD 69, VCF Volatile Compounds in Food 16.1) or by comparing only the mass spectra (MS). An asterisk (*) in a row represents significant differences between mean values at *p* < 0.05 obtained by one-way ANOVA and least significant difference (LSD) test.

**Table 2:** List of volatile aroma compounds found in monovarietal Protected Designation of Origin (PDO) and commercial-blended extra virgin olive oils by headspace solid-phase microextraction combined with comprehensive two-dimensional gas chromatography-mass spectrometry (HS-SPME-GC×GC-TOF-MS) sorted by descending Fisher *F*-ratio, compound class, retention index (monodimensional column), and semi-quantitative values in μg/kg relative to internal standard.

Compounds	Class	LRI_lit_	LRI_cal_	*F*-Ratio	Class	
					Monovarietal	Commercial/Blended
Acetic acid	Acid	1430	1422	131.84	41,378.57	608,832.20 *
3-Methyl-3-buten-1-ol	Alcohol	1250	1240	106.60	1688.17	23,649.01 *
3-Hydroxy-2-butanone	Ketone	1282	1285	90.63	83,167.97	708,294.36 *
1-Octen-3-ol	Alcohol	1412	1422	88.30	2574.32	39,706.39 *
Curcumene	(Sesqui)terpene	1288	1280	77.95	898,857.67 *	5177.70
Octanal	Aldehyde	1284	1288	77.95	998,857.69 *	5458.86
Limonene	(Sesqui)terpene	1185	1181	75.85	129,158.57	608,832.20 *
Hexyl acetate	Ester	1264	1259	70.83	6,599,680.79 *	6310.99
1-Penten-3-ol	Ketone	1308	1306	69.95	3,577,557.14 *	484,727.96
Amine n.i. ^#^			1363	67.39	22,153.18	251,515.59 *
Isoamyl alchol	Alcohol	1205	1198	66.96	318,293.21	2,312,851.79 *
2-Methyl-4-cyclohexene ^#^			1734	58.61	1803.80	45,944.97 *
2-Propanone	Ketone	1284	1294	58.11	12,487.26	523,313.68 *
Ester n.i.^#^			1509	57.56	6219.40	228,814.46 *
1-Hepten-3-ol	Alcohol	1433	1421	55.66	563,027.24	4,629,899.09 *
1-Ethyl-2-methyl-benzene	Benzeoid	1270	1258	50.71	4110.12	1,119,876.52 *
2-Hexanol	Alcohol	1226	1223	45.46	5,769,336.31 *	9376.97
Ethyl hexanoate	Ester	1236	1234	41.26	29,284.40	344,293.49 *
Isoamyl alcohol	Alcohol	1209	1213	40.93	10,846.43	70,256.03 *
Ocimene	(Sesqui)terpene	1245	1240	39.75	690,396.59 *	15,647.62
Prenal	Aldehyde	1199	1191	37.43	20,683.38	115,270.66 *
Methyl β-methylcrotonate	Ester	1148	1154	35.84	2395.85	78,223.49 *
Styrene	Hydrocarbon	1250	1247	35.70	1869.71	41,640.74 *
(*Z*)-3-Penten-1-ol	Alcohol	1307	1297	35.40	2788.20	13,671.26 *
(*Z*)-2-Pentenal	Aldehyde	1115	1109	35.24	6181.93	229,444.57 *
Protoanemonine	Lacton	1560	1570	32.50	122,469.33 *	7190.78
Valencene	(Sesqui)terpene	1689	1699	32.23	144,338.84 *	4953.13
1,3,6-Heptatriene ^#^			1899	31.30	13,879.77	70,256.03 *
(*Z*)-3-Hexenyl acetate	Ester	1312	1303	30.24	5,114,574.62 *	95,741.68
4-Cyclononen-1-ol ^#^			2486	28.89	78,002.49	293,135.33 *
Carboxaldehyde ^#^			1712	28.84	14,287.60	162,555.65 *
3,7-Dimethyl-1-octanol	Alcohol	1245	1238	28.84	332,962.19	8,020,636.63 *
4-Methyl-phenol	Phenol	2079	2076	28.73	19,100.40	61,672.58 *
Bicyclo [4.2.0]octa-1,3,5-triene ^#^			1337	28.63	0.00	183,495.37 *
3-Hexen-1-ol	Alcohol	1384	1369	28.60	14,977.65	162,555.65 *
(*Z*)-2-Penten-1-yl acetate ^#^			1210	27.60	10,072.53	83,072.51 *
Ester n.i.^#^			1335	27.50	1353.68	14,751.55 *
3-Methylpentanoate	Ester	1489	1500	26.78	1078.36	13,195.71 *
(*Z*)-2-Hexenal	Aldehyde	1120	1117	26.28	17,580,448.97 *	79,490.84
2-Methyl-1-penten-3-ol ^#^			1240	26.16	5507.31	21,808.06 *
Methylecyclooctene-3,4-diol ^#^			1896	26.14	61.05	3562.17 *
Ocimene	(Sesqui)terpene	1291	1284	25.98	10,321.31	85,200.46 *
Pentyl isobutyrate	Ester	1237	1254	25.74	38,917.41 *	26,987.41
Isoamyl acetate	Ester	1108	1107	25.37	25,476.74	108,410.01 *
3-Ethyl-1,5-octadiene	Hydrocarbon	1027	1094	24.52	288,269.98 *	63,403.01
2-Phenylethanol	Alcohol	1919	1923	24.42	2,356,534.13	4,436,340.01 *
(*Z*)-3-Hexen-1-ol	Alcohol	1387	1380	24.21	8,419,114.15 *	1,690,846.47
2-Methylbutyl acetate	Ester	1114	1109	23.80	123,593.44	678,518.54 *
5-Hexen-2-one ^#^			1520	23.80	152.64	188,184.38 *
*m*-Xylene	Hydrocarbon	1120	1116	23.16	271,361.98	801,814.21 *
2-Heptanone	Ketone	1160	1161	22.68	44,632.84	229,444.57 *
Dimethyl-1,3,5,7-octatetraene ^#^			1421	21.70	431,163.75 *	17,677.81
3-octen-2-one	Ketone	1388	1382	20.34	11,311.04	51,531.63 *
Methylsulfonylmethane	Sulfur	1890	1890	20.03	76,235.32	392,322.15 *
2-Furanmethanol	Alcohol	1659	1653	19.55	12,092.65 *	1737.38
Propylhydrazonealdehyde ^#^			1191	19.45	25,103.83	153,832.39 *
2-Methylenecyclohexanol ^#^			2450	19.23	19,701.77 *	405.28
Aldehyde n.i.^#^			1150	18.48	80,847.43 *	5697.35
[*S*-(R*,R*)]-2,3-butanediol	Alcohol	1548	1546	18.47	834.54	31,576.19 *
1-Heptanol	Alcohol	1440	1424	18.32	229,133.39	489,320.13*
*p*-Ethyltoluene	Benzeoid	1208	1208	18.30	604,528.11 *	3697.81
(*Z*)-2-Hexen-1-yl acetate	Ester	1321	1319	18.08	3572.24	87,445.13 *
Amyl acetate	Ester	1169	1158	17.44	9659.86	37,325.80 *
4-Hydroxy 2-pentenoic acid	Lactone		1663	17.22	19,924.12 *	1578.65
Monoterpene n.i.^#^			1330	16.44	80,557.56 *	6891.90
5-Hexenoic acid ^#^			1761	16.40	5732.67	41,172.27 *
Methyl pyruvate	Ester	1217	1237	16.14	47,523.10	112,112.44 *
Benzenoid n.i.^#^			1329	15.27	71,087.15	195,957.32 *
Ethyl benzoate	Benzeoid	1660	1658	14.77	156,018.91	488,215.74 *
(*E*)-Epoxy-ocimene	(Sesqui)terpene	1476	1465	14.44	27,390.24 *	6442.21
2-Methyl-4-pentenal	Aldehyde	1141	1120	14.15	1908.99	104,431.18 *
Copaene	(Sesqui)terpene	1460	1468	14.10	1,117,994.55 *	80,281.16
2-Methoxy-phenol	Phenol	1830	1835	14.00	83,229.22	225,226.46 *
Dodecane	Alkan	1200	1193	14.00	1,762,590.66 *	26,611.62
3-Methyl-2-pentanone	Ketone	1012	1016	13.76	29,918.16 *	257.94
Prenyl acetate	Ester	1251	1243	13.58	1337.15	14,792.50 *
Neo-allo-ocimene	(Sesqui)terpene	1369	1376	13.49	638,240.82 *	202,839.74
2-(acetylmethyl)-(+)-3-carene ^#^			1890	13.41	5272.02 *	166.82
*α*-Pyronene	Hydrocarbon	1365	1366	13.40	367,598.19 *	108,884.71
5-Methylfurfural	Furan	1550	1558	12.95	8333.39 *	0.00
Methyl 3-hydroxybutanoate	Ester	1461	1461	12.69	6077.46	21,076.30 *
Propanoic acid	Acid	1525	1517	12.52	953,828.13	1,898,014.73 *
2-Methyl-2-butenoic acid#			1812	11.72	4331.16 *	216.27
2-Ethyl-1-hexanol	Alcohol	1489	1484	11.64	293,625.77 *	5390.86
(*Z*)-2-Heptenal	Aldehyde	1319	1324	11.38	431,923.73	800,135.47 *
Isocumene	(Sesqui)terpene	1196	1197	11.26	25,262.49	84,791.28 *
Ester n.i.#			1293	10.65	18,124.76	150,379.31 *
Heptanal	Aldehyde	1180	1163	10.20	37,747.26	93,699.89 *
Cyclopropylbenzene	Benzeoid	1377	1361	10.04	34,705.29	71,891.03 *
Dodecanoic acid	Acid	2509	2508	9.74	986.62	140,612.14 *
Nonanoic acid	Acid	2192	2211	9.74	975.23	14,462.14 *
(*E*,*E*)-2,4-Heptadienal	Aldehyde	1480	1452	9.59	1,438,596.07	3,620,983.61 *
*α*-Ocimene	(Sesqui)terpene	1245	1235	9.37	431,193.48 *	160,245.15
Nonanal	Aldehyde	1374	1375	9.28	5,890,979.35	9,656,470.33 *
(*Z*)-2-Penten-1-ol	Alcohol	1296	1306	9.27	39,568.33	132,475.15 *
1-Octanol	Alcohol	1559	1554	9.23	10,512.66	121,637.59 *
(*E*)-2-Hexen-1-ol	Alcohol	1388	1379	9.10	398,863.62 *	16,242.32
Hexanoic acid	Acid	1880	1881	8.93	29,882.90 *	1,798.32
(*Z*)-2-Penten-1-ol	Alcohol	1320	1316	8.82	6,095,917.32 *	24,624.06
1-Penten-3-one	Ketone	1038	1096	8.73	54,514.73 *	1,594.20
2-Octanol	Alcohol	1398	1402	8.62	61,420.45	221,737.82 *
Prunolide	(Sesqui)terpene	2048	2052	8.61	103,712.87 *	21,671.55
Allo-ocimene	(Sesqui)terpene	1369	1370	8.58	61,726.03 *	2328.21
3,7-Dimethyl-1,6-octadiene	Hydrocarbon	1050	1049	8.51	4922.38 *	879.32
Decanoic acid	Acid	2278	2267	8.33	7316.34 *	2015.68
2-Ethyl-furan	Furan	960	950	8.22	6844.34	31,477.56 *
3-Pentanol	Alcohol	1106	1099	8.17	48,766.06	529,880.55 *
Ethyl tiglate	Ester	1232	1236	8.00	136,021.23 *	7618.67
(*E*)-2-Hepten-1-ol	Alcohol	1507	1499	7.99	1801.99	94,353.20 *
4-Penten-1-ol	Alcohol	1290	1295	7.73	5902.15	1048.98 *
Ethyl 3-furoate ^#^			1609	7.71	0.00	3666.65 *
2-Pentanol	Alcohol	1117	1104	7.43	30,503.88	249,804.69 *
Acetophenone	Ketone	1627	1645	7.42	65,342.62 *	0.00
Farnesene	(Sesqui)terpene	1755	1745	7.24	110,230,278.27 *	160,673.90
Toluene	Hydrocarbon	1055	1049	7.22	136,021.23 *	7618.67
4-Ethyl-benzaldehyde	Benzeoid	1728	1726	6.87	13,952.89 *	4618.86
Methyl hexanoate	Ketone	1177	1165	6.62	35,131.15	89,231.51 *
*α*-Muurolene	(Sesqui)terpene	1728	1708	6.38	578,172.46 *	257,509.06
2,4-Hexadien-1-ol	Alcohol	1523	1502	6.36	13,201.54 *	928.53
(*E*)-2-Penten-1-ol	Alcohol	1321	1320	6.22	103,370,445.14 *	3991.97
(*Z*)-Bergamotene	(Sesqui)terpene	1909	1899	6.19	13,104.79 *	766.76
(*E*)-2-hexenal	Aldehyde	1196	1195	6.18	26,108,383.11 *	510,524.92
2-Ethyl-1,3-dimethyl-benzene	Benzeoid	1347	1344	6.14	52,113.37	114,494.73 *
5-Ethyl-2(5*H*)furanone	Furan	1733	1734	6.10	7,784,864.23 *	3779,623.65
3-Penten-2-one	Ketone	1111	1109	5.94	11,013.36	19,977.38 *
2-Pentyl-furan	Furan	1215	1213	5.71	118,791.62 *	15,014.39
1-Butanol	Alcohol	1139	1139	5.54	595,215.31 *	130,420.18
2-Octenal	Aldehyde	1416	1410	5.42	52,163.93	97,028.48 *
(*Z*)-2-Hexen-1-ol	Alcohol	1436	1421	5.41	246,873.49 *	2877.40
Cumene	(Sesqui)terpene	1288	1280	5.40	248,241.74	447,051.04 *
Citronellol	(Sesqui)terpene	1757	1754	5.24	24,356.34 *	14,352.34
4-Ethyl-*m*-xylene	Hydrocarbon	1319	1323	5.22	63,423.72	121,964.53 *
*m*-Ethylmethylbenzene	Benzeoid	1246	1247	5.13	0.00	14,536.13 *
Methyl (*Z*)-3-hexenoate	Ester	1948	1941	5.05	3259.45	10,258.45 *
Hexyl butanoate	Ester	1388	1401	4.91	40,413.52 *	16,743.01
5-Methyl-2(3*H*)-furanone	Furan	1416	1412	4.84	47,851.94	21,192.70 *
Ethyl 2-butenoate	Ester	1161	1151	4.74	0.00	4134.40 *
Sulcatone	Hydrocarbon	1338	1329	4.74	4060.19	408,627.09 *
Isobutanoic acid	Acid	1565	1554	4.55	4937.86	412,112.44 *
4,8-Dimethyl-1,3,7-nonatriene	Hydrocarbon	1304	1289	4.38	12,734,050.10 *	6165,191.22
Benzaldehyde	Aldehyde	1518	1508	4.32	560,180.99 *	25,052.60
2-Methyl-3-penten-1-ol	Alcohol	1354	1350	4.24	69,968.019 *	21,918.81
(*E*)-3-Hexen-1-ol	Alcohol	1378	1370	4.16	812,8013.46	11,029,551.14 *
(*E*)-2-Hexenoic acid	Acid	1962	1955	n.s	2,467,127.31	2,652,972.27
(*E*)-3-Hexenyl acetate	Ester	1333	1329	n.s	5,281,303.89	6227,652.94
α-Copaene	(Sesqui)terpene	1460	1468	n.s	32,609.69	14,152.46
β-Ocimene	(Sesqui)terpene	1255	1245	n.s	6512.82	10,863.14
1-Hexanol	Alcohol	1336	1337	n.s	128,095.59	104,703.26
1-Hexen-3-ol	Alcohol	1230	1225	n.s	25,697.59	16,238.11
1-Pentanol	Alcohol	1245	1243	n.s	715,152.52	560,511.12
2,4-Pentadienal	Aldehyde	1197	1207	n.s	332,491.61	273,823.24
(*E*)-2-Pentenal	Aldehyde	1121	1111	n.s	661,208.23	841,871.57
3-Hexanal	Aldehyde	1146	1120	n.s	517,211.48	594,193.33
(*E*)-3-Hexenyl butanoate	Ester	1451	1449	n.s	99,944.21	54,576.52
δ-Cadinene	(Sesqui)terpene	1729	1726	n.s	26,908.50	16,020.85
Octanoic acid	Acid	2071	2057	n.s	21,213.91	34,572.98
Pentanoic acid	Acid	1720	1716	n.s	212,384.72	325,869.83
Pentanol	Alcohol	1271	1261	n.s	1734.76	1069.51

Identification: the volatile aroma compounds were identified on the basis of the comparison of their mass spectra with those from a mass spectral database, as well as by retention indexes matches on a similar phase column (NIST Chemistry WebBook SRD 69, VCF Volatile Compounds in Food 16.1), except compounds designated by ^#^—tentatively identified by mass spectra database matches. LRIlit—linear retention index from the literature, LRIexp—linear retention index. An asterisk (*) in a row represents significant differences between mean values at *p* < 0.05 obtained by one-way ANOVA and least significant difference (LSD) test.

**Table 3:** List of phenols found in monovarietal Protected Designation of Origin (PDO) and commercial-blended extra virgin olive oils by high-performance liquid chromatography with diode-array and mass spectrometric detection (HPLC-DAD-ESI/MS) sorted by descending Fisher *F*-ratio, phenol class, and concentration (mg/kg). An asterisk (*) in a row represents significant differences between mean values at *p* < 0.05 obtained by ANOVA and least significant difference (LSD) test.

Compounds	*F*-Ratio	Class	
		Monovarietal	Commercial-Blanded
Acetoxypinoresinol	6.06	16.437 *	10.537
Pinoresinol	5.89	5.964 *	3.850
*p*-HPEA-EDA	5.31	28.816	45.282 *
Hydroxytyrosol	n.s	13.253	12.906
Tyrosol	n.s	17.873	16.497
Vanillic acid	n.s	0.735	0.726
3,4-DHPEA-EDA I	n.s	15.846	22.012
Oleuropein	n.s	10.912	11.736
3,4-DHPEA-EDA II	n.s	1.087	1.448
3,4-DHPEA-EA	n.s	11.759	15.871
*p*-HPEA-EA	n.s	16.451	11.943
Vanillin	n.s	0.374	0.339
*p*-Coumaric acid	n.s	0.437	0.377

## Supplementary Materials

The following are available online, Figure S1: Chromatograms of volatile aroma compounds found in monovarietal PDO and commercial-blended extra virgin olive oil by headspace solid-phase microextraction combined with gas chromatography/mass spectrometry (HS-SPME-GC-MS). Figure S2: Contour plots (2D-chromatograms) of volatile aroma compounds found in a) monovarietal PDO and commercial-blended extra virgin olive oils by headspace solid-phase microextraction combined with comprehensive two-dimensional gas chromatography-mass spectrometry (HS-SPME-GC×GC-TOF-MS). Figure S3: Chromatograms of phenols found in a) monovarietal PDO and b) commercial-blended extra virgin olive oils by high-performance liquid chromatography with UV at wavelength of 280 nm and 310 nm respectively. Figure S4: Principal component analysis (PCA) of HPLC-DAD-MS data; (a) indicates the score plot with all compounds; (b) loading plot correlated with sensory attributes in EVOO samples. Table S1: Standards used for all the analysis
