# Supplementary material for: Complementary Untargeted and Targeted Metabolomics for Differentiation of Extra Virgin Olive Oils of Different Origin of Purchase Based on Volatile and Phenolic Composition and Sensory Quality
Source: Molecules. 2019 Aug 9;24(16):2896. doi: 10.3390/molecules24162896 (PMC6720806; doi:10.3390/molecules24162896)
Supplement: Supplementary file 1 [file molecules-24-02896-s001.zip › supplementary/Figures S1_S2_S3_S4.pdf]

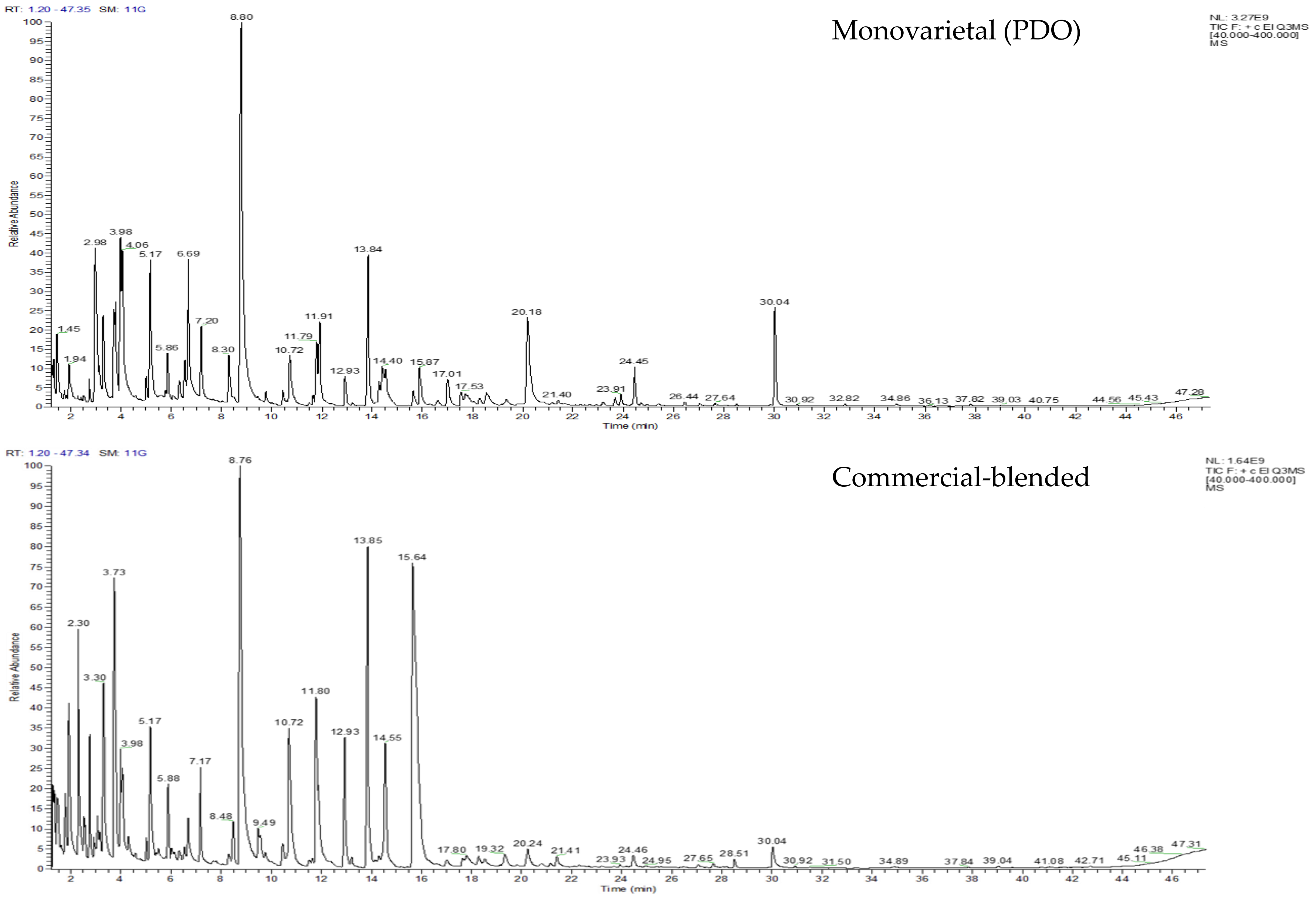

Figure S1: Chromatograms of volatile aroma compounds found in monovarietal PDO and commercial-blended extra virgin olive oil by headspace solid-phase microextraction combined with gas chromatography / mass spectrometry (HS-SPME-GC-MS/MS).

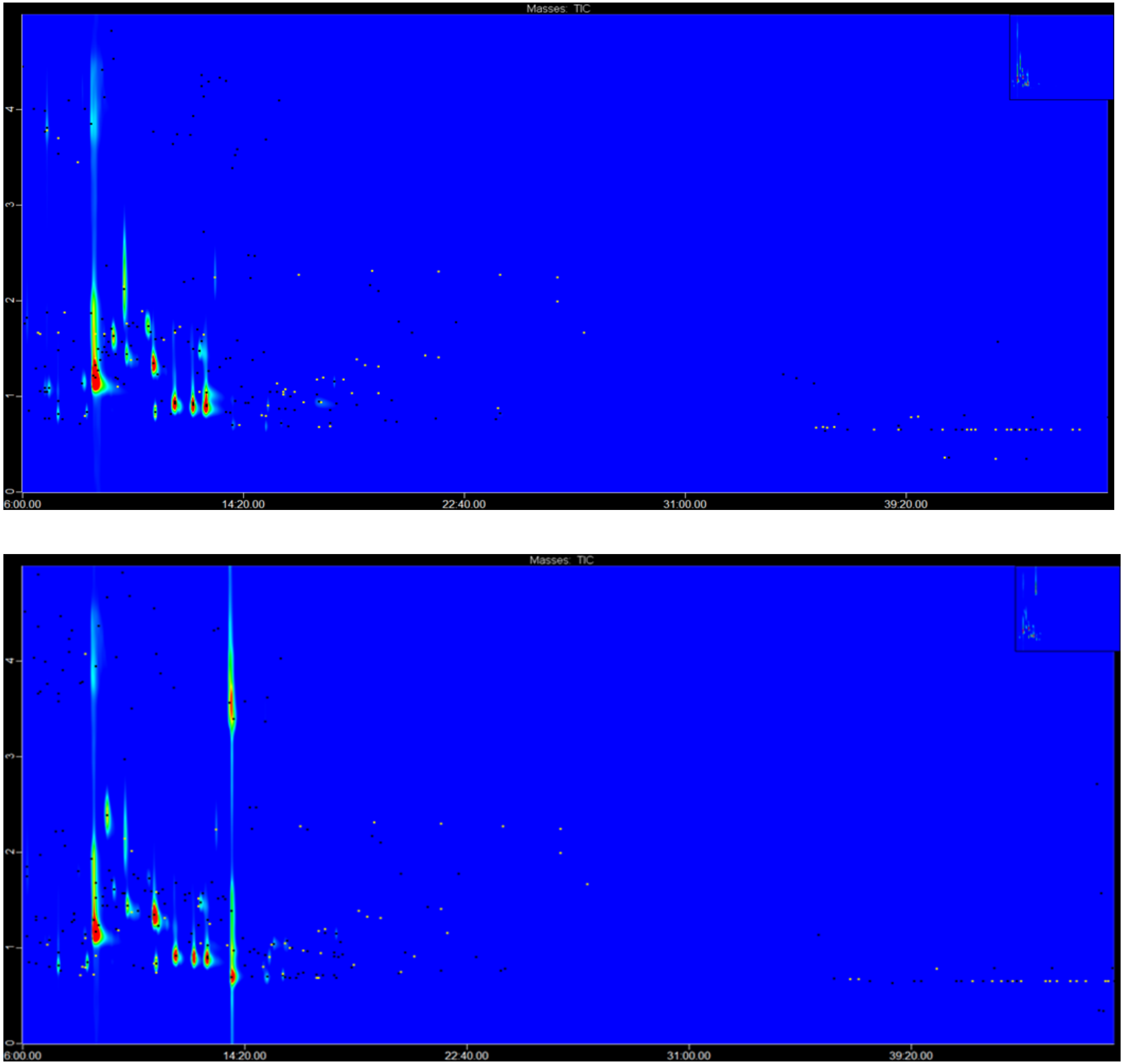

Monovarietal (PDO)

Commercial-blended

Figure S2: Contour plots (2D-chromatograms) of volatile aroma compounds found in monovarietal PDO and commercial-blended extra virgin olive oils by headspace solid-phase microextraction combined with comprehensive two-dimensional gas chromatography-mass spectrometry (HS-SPME-GC×GC-TOF-MS).

Monovarietal (PDO)

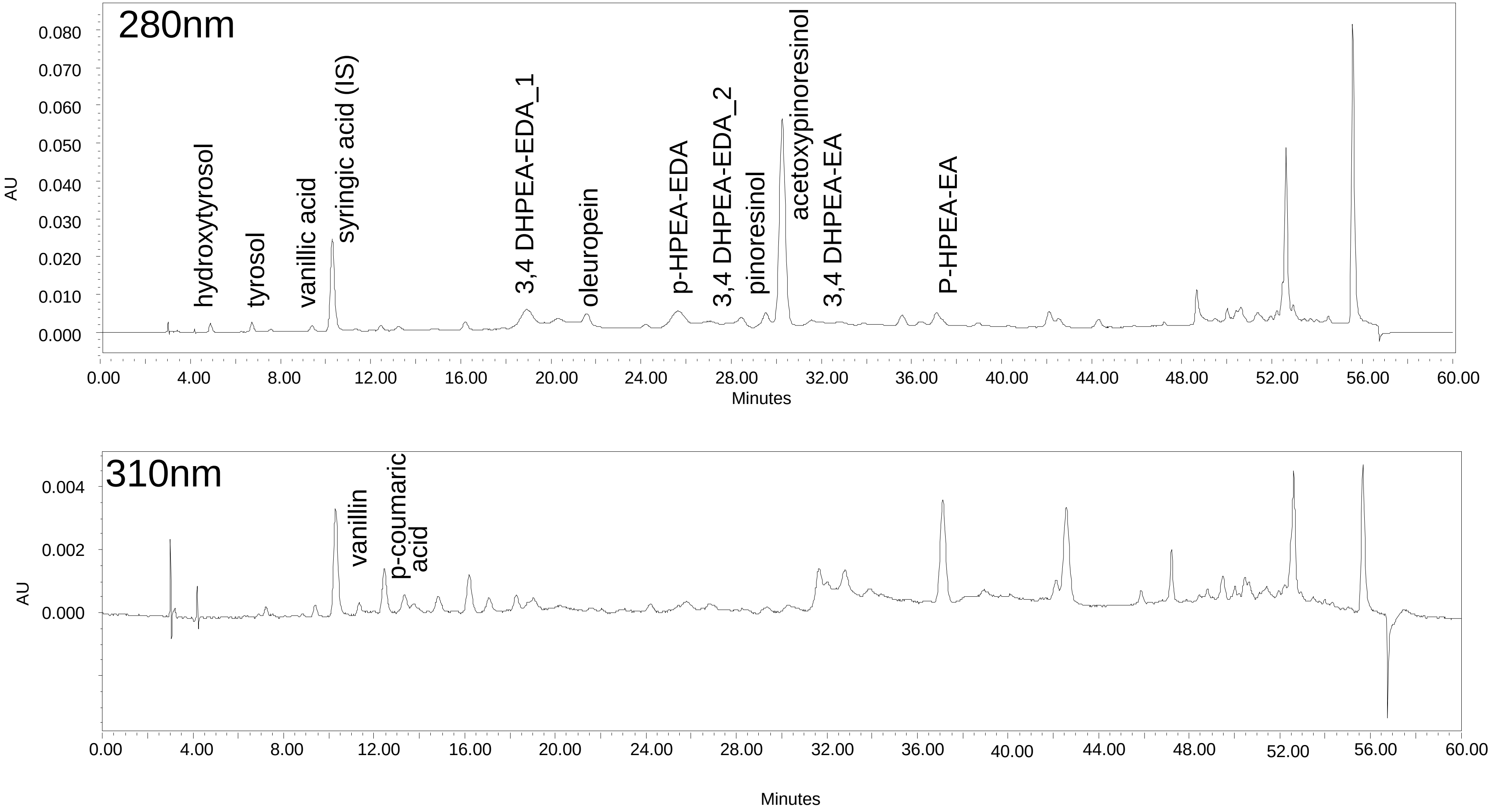

Figure S3: Chromatograms of phenols found in monovarietal PDO and commercial-blended extra virgin olive oils by high-performance liquid chromatography with UV at wavelength of 280 nm and 310 nm respectively.

Commercial-blended

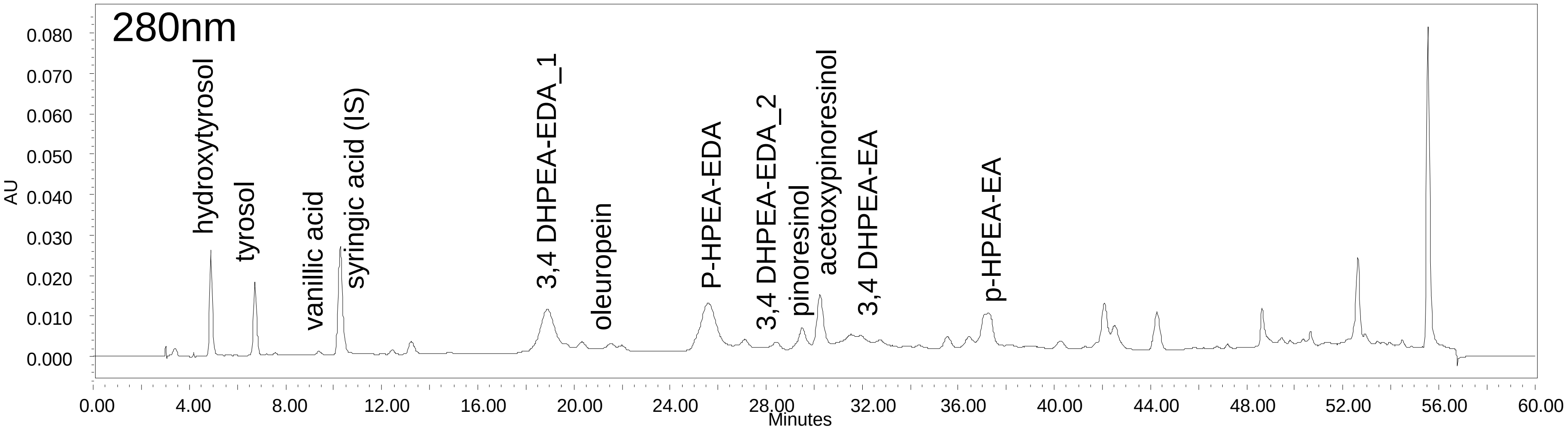

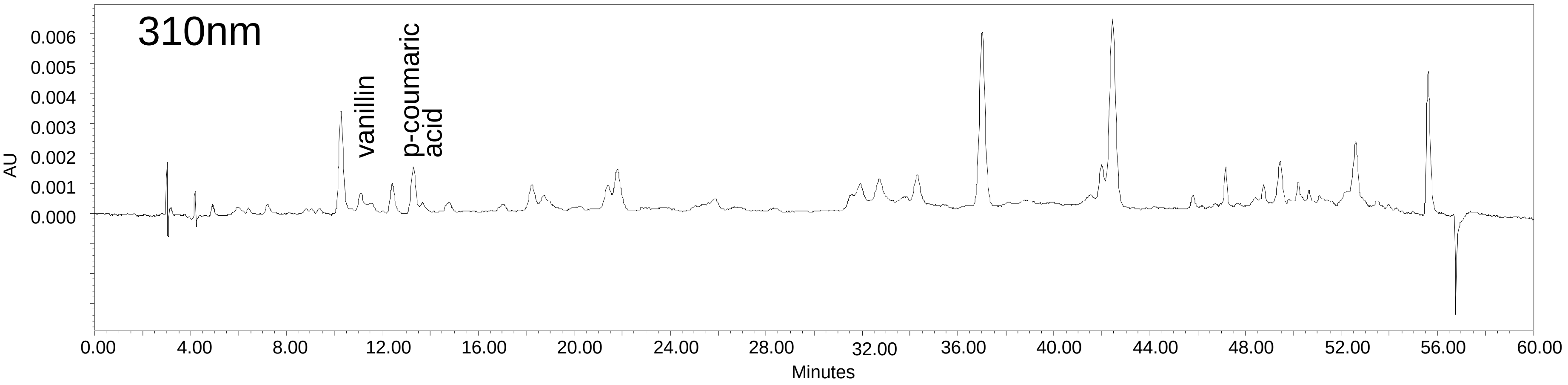

Figure S3: Chromatograms of phenols found in monovarietal PDO and commercial-blended extra virgin olive oils by high-performance liquid chromatography with UV at wavelength of 280 nm and 310 nm respectively.

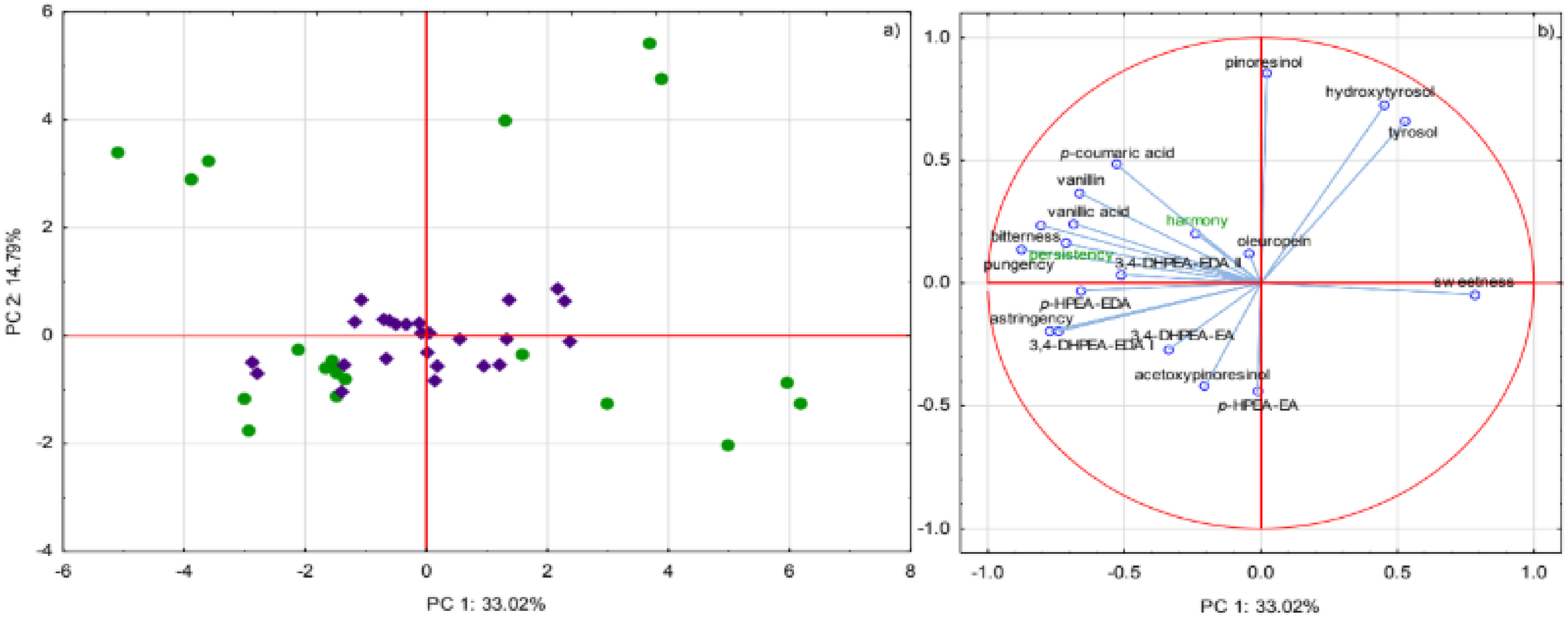

Figure S4: Principal component analysis (PCA) of HPLC-DAD-MS data; (a) indicates the score plot with all compounds; (b) loading plot correlated with sensory attributes in EVOO samples
