# Supplementary material for: Complementary Untargeted and Targeted Metabolomics for Differentiation of Extra Virgin Olive Oils of Different Origin of Purchase Based on Volatile and Phenolic Composition and Sensory Quality
Source: Molecules. 2019 Aug 9;24(16):2896. doi: 10.3390/molecules24162896 (PMC6720806; doi:10.3390/molecules24162896)
Supplement: Supplementary file 1 [file molecules-24-02896-s001.zip › supplementary/Table S1.pdf]

Table S1. Standards used for all the analysis

Class	Compound	CAS n°	Chemical formula	Furnisher
Acids				
	3-methylbutanoic acid	503-74-2	C5H10O2	Sigma
	Acetic acid	64-19-7	C2H4O2	Sigma
	Butanoic acid	107-92-6	C4H8O2	Sigma
	Formic acid	64-18-6	CH2O2	Fluka
	Heptanoic acid	111-14-8	C7H14O2	Sigma
	Hexanoic acid	142-62-1	C6H12O2	Fluka
	Nonanoic acid	112-05-0	C9H18O2	Aldrich
	Octanoic acid	124-07-2	C8H16O2	Aldrich
	Pentanoic acid	109-52-4	C5H10O2	Aldrich
	Propionic acid	79-09-4	C3H6O2	Aldrich
	Sorbic acid	110-44-1	C6H8O2	Sigma
Alcohols				
	1-hexanol	111-27-3	C6H14O	Aldrich
	1-nonanol	143-08-8	C9H20O	Aldrich
	1-octanol	111-87-5	C8H18O	Aldrich
	(E)2-hexen-1-ol	928-95-0	C6H12O	Sigma
	(Z)3-hexen-1-ol	928-96-1	C6H12O	Sigma
	3-methyl-1-butanol	123-51-3	C5H12O	Sigma
	Benzene methanol	100-51-6	C7H8O	Fluka
	Ethanol	64-17-5	C2H6O	Sigma
	Menthol	2216-51-5	C10H20O	Sigma
	Phenylethyl alchol	60-12-8	C8H10O	Aldrich
Aldeydes				
	(E)1,2-pentenal	1576-87-0	C5H8O	Sigma
	2,4-hexadienal	80466-34-8	C6H8O	Sigma
	(E)2-hexenal	6728-26-3	C6H10O	Aldrich
	2-methyl-butanal	96-17-3	C5H10O	Fluka
	Benzaldehyde	100-52-7	C7H6O	Fluka
	(Z)3-hexenal	6789-80-6	C6H10O	Fluka
	Hexanal	66-25-1	C6H12O	Sigma
	Nonanal	124-19-6	C9H18O	Sigma
	Octanal	124-13-0	C8H16O	Sigma
	Pentanal	110-62-3	C5H10O	Sigma
Ketones				
	1-penten-3-one	1629-58-9	C5H8O	Sigma
	3-pentanone	96-22-0	C5H10O	Sigma
	4-methyl-2-pentanone	108-10-1	C6H12O	Sigma
Esters				
	(E)2-hexen-1-ol acetate	2497-18-9	C8H14O2	Alderich
	(Z)3-hexen-1-ol acetate	3681-82-1	C8H14O2	Alderich
	Ethyl acetate	141-78-6	C4H8O2	Sigma
	Ethyl benzene	93-89-0	C9H10O2	Sigma

Hexyl acetate	142-92-7	$C_8H_{16}O_2$	Sigma
Phenols			
2-methoxy phenol	90-05-1	$C_7H_8O_2$	Sigma
OH-tyrosol	10597-60-1	$C_8H_{10}O_3$	Sigma
Tyrosol	501-94-0	$C_8H_{10}O_2$	Sigma
Vanillin	121-33-5	$C_8H_8O_3$	Sigma
p-coumaric acid	501-98-4	$C_9H_8O_3$	Sigma
Oleuropein	32619-42-4	$C_{25}H_{32}O_{13}$	Sigma
Pinoresinol	487-36-5	$C_{20}H_{22}O_6$	Sigma
Vanillic acid	121-34-6	$C_8H_8O_4$	Sigma
Phenol	108-95-2	C_6H_6O	Sigma
Hydrocarbons			
β-ocimene	13877-91-3	$C_{10}H_{16}$	Sigma
Copaene	3856-25-5	$C_{15}H_{24}$	Sigma
Limonene	138-86-3	$C_{10}H_{16}$	Sigma
Octane	111-65-9	C_8H_{18}	Sigma
Valencene	4630-07-3	$C_{15}H_{24}$	Alderich
